# An Exploration of Responses to Drug Conditioned Stimuli during Treatment for Substance Dependence

**DOI:** 10.1155/2013/394064

**Published:** 2013-08-26

**Authors:** Benjamin Goddard, Leanne S. Son Hing, Francesco Leri

**Affiliations:** Department of Psychology, University of Guelph, 50 Stone Road East, Guelph, ON, Canada N1G 2W1

## Abstract

Although it is well established that drug conditioned stimuli produce a variety of conditioned responses, it is not known whether such stimuli can also reinforce an arbitrary operant response and thus serve as conditioned reinforcers. Volunteers (*n* = 39) recruited from a residential treatment center for substance dependence were tested on a task in which presses on computer keys activated images of drugs/drug paraphernalia on a progressive ratio schedule of reinforcement. They also completed a personalized craving questionnaire and a personalized Implicit Association Test. A significant bias in responding was found for images of preferred drugs/route of drug administration. Craving, however, was low and the images generated negative evaluative reactions. Two additional studies were performed to ascertain the generalizability of the effects to a different population of drug-using individuals (i.e., students who drink) and to incentive stimuli of a different nature (i.e., sexual). The additional studies partially replicated and extended the central findings of the main study. Therefore, although these data should be considered preliminary in light of small group sizes, it is concluded that cue specificity and availability of the unconditioned stimuli (drugs and sex) plays a role in modulating responding maintained by conditioned reinforcers.

## 1. Introduction

Drug conditioned stimuli, which can be discrete (i.e., a syringe) and/or environmental (i.e., a room), acquire the ability to activate drug-oriented behaviors because they are repeatedly perceived in conjunction with the unconditioned effects of drugs of abuse [1–3]. Hence, through Pavlovian conditioning, drug conditioned stimuli become wanted [4] and preferred [5], grab attention [6–8], and produce a variety of physiological and psychological responses [9–15].

The current study had two primary objectives. The first was to establish whether drug conditioned stimuli (i.e., images of drugs and drug paraphernalia) can serve as conditioned reinforcers. Conditioned reinforcing stimuli, unlike primary reinforcing stimuli, strengthen behavioral responses in virtue of their learned value. Therefore, the objective of this study was to determine whether the occurrence of stimuli associated with the effects of drugs can maintain an arbitrary operant response (i.e., pressing a computer key) in the absence of drugs [16]. This is of interest because it is possible that the assessment of the reinforcing value of drug conditioned stimuli could complement other measures of “cue reactivity” such as self-reported craving [17, 18], and thus help predict clinical outcomes [19, 20].

To determine whether drug conditioned stimuli would reinforce arbitrary operant responses, subjects were recruited from Stonehenge Therapeutic Community, a long-term (6 months) residential treatment facility designed for chronic and relapsing substance dependence. Therefore, these individuals were likely to have experienced substantial conditioning as a result of excessive exposure to various drugs of abuse. Although the selection of this population precluded manipulation of important variables such as availability of the unconditioned stimuli (i.e., drugs), it allowed the exploration of whether this novel putative index of cue-reactivity could be related to self-reported drug cravings, and predictive of treatment completion, which is typically low in therapeutic communities [21]. 

The second objective was to study the relationship between explicit behavioral reactivity (i.e., operant responding) and automatic evaluative processes elicited by drug conditioned stimuli. Using the Implicit Association Test (IAT), for example, it has been established that words such as beer, wine, whisky, and rum generate significant automatic negative responses in heavy drinking individuals who do not try to abstain [22]. That is, subjects are faster at categorizing alcohol-associated words with negative concepts such as “bad” or “disgust,” than with positive concepts such as “good” or “pleasant.” In light of leading neurobiological theories of addiction predicting dissociations between what people do when they are exposed to drug associated stimuli compared to how they feel [23, 24], the IAT was used to assess automatic responses to drug conditioned stimuli within the context of their conditioned reinforcing effect. It was hypothesized that the two measures would reveal independent aspects of cue reactivity.

Studies performed in clinical populations, however, can have limited generalizability. That is, it can often be questioned whether the results apply to other clinical populations, or if they can help understand basic psychological processes that play a role in the behavior of nonclinical samples. Because the third objective of this research was to explore the relationship between basic psychological processes, two additional studies were performed. These studies were specifically implemented in nonmatched groups to ascertain whether significant relationships could be observed between cravings for incentives, behavioral responses to stimuli-associated with these incentives, and automatic evaluative processes elicited by these stimuli.

 Therefore, one study investigated whether drug conditioned stimuli can reinforce operant behavior also in individuals who regularly consume drugs, but are not dependent and not in treatment for excessive use. Therefore, volunteers were recruited from the population of undergraduate students at the University of Guelph on the basis of self-reported levels of alcohol consumption. The focus on this particular drug was constrained by the selection of the sample: undergraduate students in this University rarely report the use of other drugs, including cannabis. 

The second study investigated whether only stimuli paired with drugs of abuse can function as conditioned reinforcers. Therefore, always in undergraduate students, it was tested whether images of sexy attractive models in swimsuits could support operant responding. Sexual stimuli were selected because: (1) it is fairly intuitive to predict the gender of the reinforcing stimulus in heterosexual individuals; (2) it is known that sexual stimuli can act as conditioned reinforcers in animals [25]; and (3) it is clear that responses to sexual stimuli can be observed in the absence of sexual “addiction” [26]. Similar methodologies were employed in the three studies to allow for meaningful comparisons across findings.

## 2. Methods

### 2.1. Participants

#### 2.1.1. Stonehenge Therapeutic Community Study

The sample consisted of 28 males and 11 females, aged (mean ± standard error of the mean; sem) 36.8 ± 1.5 and 37.5 ± 3.9, respectively, primarily Caucasian (87%), with education below university level (98%). The power calculation was performed using the effect size estimated using the *Cohen's d* model, although subsequent analyses required to split the sample in subgroups (see below). The vast majority (92%) of subjects had received previous treatment; 38% reported one, and 54% reported 2 or more treatment attempts in different programs. All subjects were poly-drug users. Excluding tobacco (because almost all smoked cigarettes), the drug most often used (more than 15 days) in the 30 days prior to treatment admission was crack/cocaine (77% of subjects). Volunteering participants were eligible only if they had completed at least two weeks of treatment. The average (±sem) number of days in treatment at the time of study interview was 70 ± 5.5. Typical duration of the entire program is between 120 and 180 days. The Research Ethics Board of the University of Guelph approved the study. 

#### 2.1.2. Additional Studies

*Alcohol Study. *The study of responses to alcohol-related stimuli included 49 participants (17 males and 32 females, aged 19.1 ± 0.2 and 19.2 ± 0.2, resp.). *Sex Study.* The study of responses to sexual stimuli included 106 heterosexual participants (43 males and 63 females, aged 18.6 ± 0.2 and 18.3 ± 0.1, resp.). All participants were undergraduate students at the University of Guelph, recruited by mass-testing questions about drinking and sexual behavior (see below). The Research Ethics Board of the University of Guelph approved both studies.

### 2.2. Measures and Procedures

#### 2.2.1. Stonehenge Therapeutic Community Study

First, a brief survey assessed drug use in the 30 days prior to arrival at the community, as well as drug of choice and preferred route of administration. 

Second, participants completed a questionnaire about craving for their drug of choice. This questionnaire included 10 questions about desire for the drug (i.e., “I have an urge for_________”) and 10 questions about anticipated drug effects (i.e., “Using ____________ right now would make me feel less tired”). The experimenter completed the blank for each item with the particular participant's drug of choice. The questions were derived from items common to half of 12 validated questionnaires assessing craving for alcohol, cocaine, speed, heroin, or tobacco [27–37]. This “composite” questionnaire was created because participants drug of choice was not known prior to the initial survey, and thus there was a need for questions that would apply regardless of the name of the drug that was used to fill the blanks. Answers were provided on a visual analog scale ranging from 0 to 10. Therefore, the maximal total craving score on this questionnaire was 200. The Cronbach's alpha of this composite craving questionnaire was 0.91.

**Figure 1:**
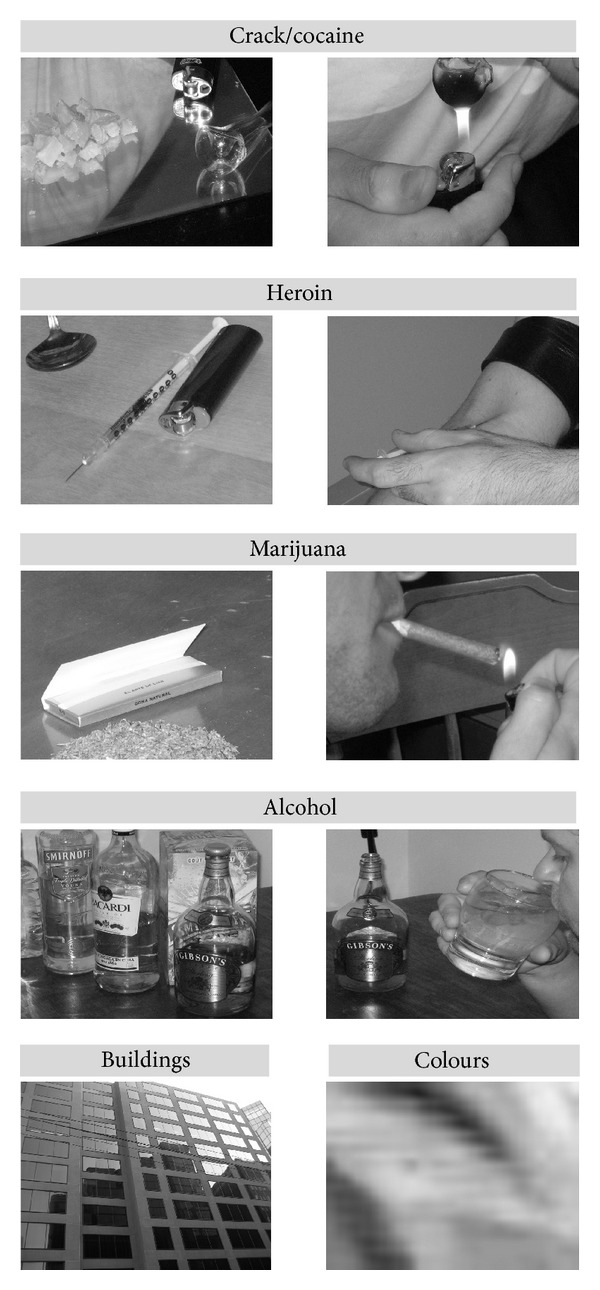
Examples of images (in black and white) employed in the CRT task.

Third, volunteers were asked to perform the Conditioned Reinforcement Task (CRT). This was an adaptation of a conditioned reinforcement procedure [38–41] in which operant behavior is reinforced by stimuli previously associated with the effects of drugs of abuse. In the current study, participants responded to keys generating pictures (see Figure 1) of drug look-alike substances (i.e., white powder, crystals), of actual drugs (i.e., bottles of different alcoholic beverages), of simulated drug taking behavior (i.e., snorting, smoking, injecting, drinking), and of drug paraphernalia (i.e., syringe, needle, crack pipe). Six keys were linked to 6 categories of images, and each category included 40 images. Four categories were created to represent drugs of choice commonly reported by individuals in treatment at Stonehenge: cocaine/crack, heroin, alcohol, and marijuana. Two additional categories were created for control purposes and included pictures of buildings, and random colours. These categories were selected because buildings are recognizable visual stimuli with neutral motivational value, and random colors can have motivational value but do not represent identifiable objects. All images were equalized for contrast and luminance.

Pressing any of the 6 keys activated a single image of a specific category for 1 second according to a progressive ratio schedule of reinforcement. The computer randomly determined the order of image presentation within each category/key. The progressive ratio schedule has been employed in animal [42] and humans [43, 44] to measure motivation to self-administer drugs when the response requirement for each subsequent administration progressively increases within the session [45]. Of course, in the current study, no drug was provided after completion of each response ratio.

Participants were not informed about the association between keys and image categories prior to the beginning of testing, no practice trials were given, and there was no time limit to perform the task. The test began after the following instructions were read:

*“Pressing the keys D, F, G, H, J, and K will produce pictures on the screen. Some of these will be drug-related and some will not. Pressing the same key twice will produce another picture and so on. Presses required will go up after each picture. You have complete choice as to which keys you choose to press. There is no requirement and you may stop at any time. Please press any key to begin.”*

Fourth, after a short break, all participants completed a personalized IAT [46–49] to assess automatic responses to drugs generated by exposure to the same drug-associated images employed in the CRT task. Unlike the traditional IAT that includes general attribute categories such as “good” and “bad,” the personalized IAT requires a categorization of test items into attributes that are specific to the individual being tested: “I like” and “I dislike.” This particular version was selected because it reduces extrapersonal automatic contamination [46, 50, 51] and thus better taps into personal automatic associations with drug (and nondrug) stimuli.

Therefore, participants were asked to categorize 40 drug associated images of their drug of choice, 40 control images (building images), 6 positive words (i.e., joy, happy), and 6 negative words (i.e., rotten, disgust) into one of four categories: two concept categories (“drugs” and “buildings”), and two attribute categories (“I like” and “I dislike”). 

The IAT consisted of 5 blocks of trials. For each trial, participants were required to sort a target word or a target picture that appeared in the middle of the screen into a category that appeared at the top left or the top right of the screen using respective computer keys. In the first practice block, participants sorted drug and control images into the concept category “buildings” on the left or “drugs” on the right. In the next practice block, participants sorted positive and negative words into the attribute category “I like” on the left, or “I dislike” on the right. The third block was a test block: the earlier tasks were combined and now participants sorted both picture and word targets in categories “buildings” combined with “I like” that appeared on the left of the screen, or categories “drugs” combined with “I dislike” that appeared on the right side of the screen. In the final two test blocks, the concept and attribute categories matches were reversed. Therefore, in the next practice block, target images were sorted into either “drugs” on the left, or “buildings” on the right side of the screen. And, in the final test block, stimuli were sorted into either categories “drugs” combined with “I like” that appeared on the left, or categories “buildings” combined with “I dislike” that appeared on the right side of the screen. The dependent measure in this task is time (msec) required to assign target words and pictures to the matched concept/attribute categories on test two blocks. Faster reaction times reflect dominant automatic associations between concept and attribute categories that share a side of the computer screen. The interesting comparison was between reaction times displayed on the test blocks when “drugs” and “I dislike” shared a side of the screen versus when the side was shared by “drugs” and “I like.”

#### 2.2.2. Additional Studies

The measures and procedure employed in the two additional studies differed from the main study in three ways. 

First, in the Alcohol study, student participants completed the alcohol dependency scale (ADS), in which a score of 9 or greater indicates potential problematic drinking [52]. They also (1) self-reported drinking in the 30 days prior to study interview (days of drinking and number of times they drank 0–4, 5–9, or 10+ standard drinks on each of those occasions); (2) completed the timeline follow-back measure (TFM) [53]; and (3) completed the questionnaire about craving with the word “alcohol” included in each question. In the Sex study, participants answered a questionnaire about aspects of sexual behavior in the 30 days prior to study interview (sexual relationship status, frequency of intercourse, and number of partners), and completed the questionnaire about craving employed in the other studies with the spaces for drug names (i.e., cocaine, alcohol) filled by the word “sex.” 

Second, in the Sex study, the images of drugs/drug use/drug paraphernalia employed in the CRT task were replaced by pictures of sexy, attractive models (women and men) in swimsuits taken from magazines such as Maxim, FHM, and GQ. Previously, the pictures were ranked on sexiness by a focus group, and the top 40 were selected for the study. Two additional control categories were included representing stimuli likely to have motivational value in undergraduate students: “junk” food (McDonald's, pizza) and snack food (chocolate, potato chips). As in the study at Stonehenge Therapeutic Community, there were also control pictures of building and random colours. The stimuli used in the Stonehenge study and in the Alcohol study were identical.

Finally, in the *Alcohol study*, participants categorized the images of alcoholic beverages and drinking (from the CRT), control images (buildings), positive words (i.e., joy, happy), and negative words (i.e., rotten, disgust), into one of four categories: two concept categories (“alcohol” and “buildings”) and two attribute categories (“I like” and “I dislike”). Similarly, in the *Sex study*, participants categorized the sexy images of opposite sex models (from the CRT), control images (buildings), positive words (i.e., joy, happy), and negative words (i.e., rotten, disgust) into one of four categories: two concept categories (“sex” and “buildings”), and two attribute categories (“I like” and “I dislike”).

### 2.3. Data Analysis

For the CRT, one-, two-, and three-factor repeated measure ANOVAs were used to compare total responding across the various keys. When data were not normally distributed, the analysis was performed using the Friedman repeated measures ANOVA on ranks. In case of significant interactions or significant main effects, multiple comparisons were performed using the Student-Newman-Keuls method to identify individual mean differences (*α* = 0.05). 

For IAT the data, mean response latencies to categorize stimuli in the critical test blocks were computed and compared using paired *t*-tests. If they were not normally distributed, the data were analyzed using the Wilcoxon signed rank test. Furthermore, an IAT Difference score was calculated for each individual with lower scores reflecting more negative-automatic attitudes toward drugs/alcohol/sex [54]. Pearson correlations were employed to explore relationships between IAT Difference scores and other variables. For all analyses, the specific values of nonsignificant findings are not reported.

For analyses presented below, subgroups were created on the basis of drug of choice and preferred route of administration. Unfortunately, for the heroin- and alcohol/oral administration-based groupings, the sample sizes were too small for statistical analyses. Therefore, data for these sub-groups are reported in descriptive terms only.

## 3. Results

### 3.1. Stonehenge Therapeutic Community Study

From the admission survey, it was determined that 31, 4, and 4 subjects identified crack/cocaine, heroin, and alcohol as their drug of choice, respectively. As a result, these three groups of subjects were employed for analysis. The overall average (±sem) level of self-reported craving was low (47 ±  6.3), with no significant differences between the groups.

**Figure 2:**
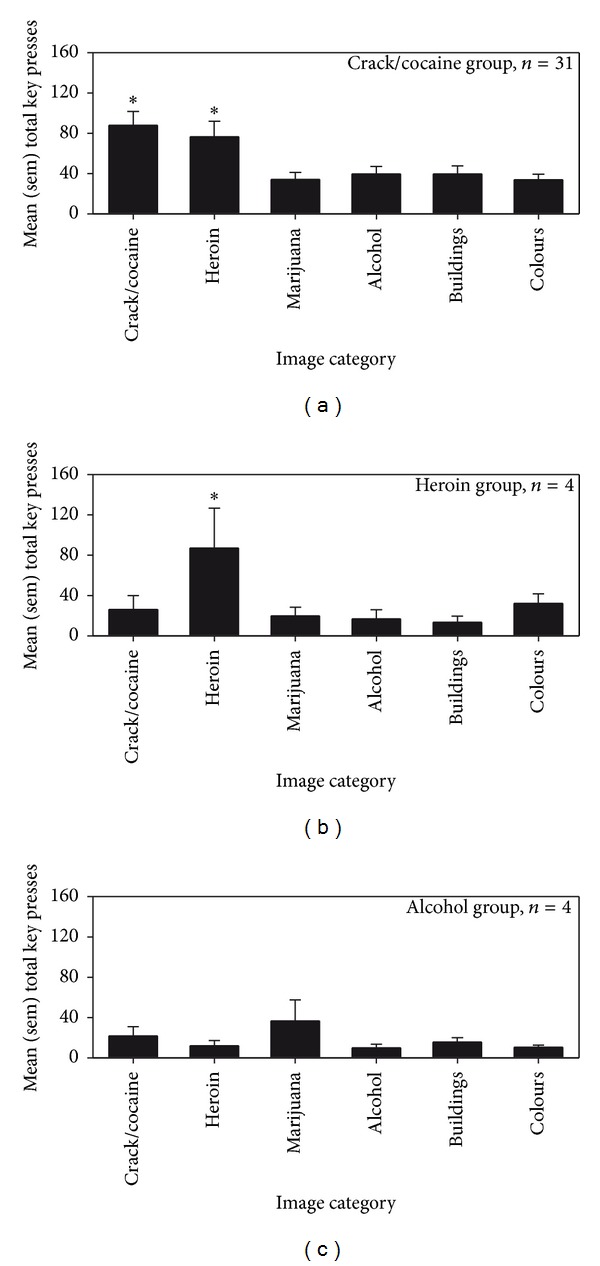
CRT performance in volunteers tested at Stonehenge Therapeutic Community. Mean and sem total number of responses made on computer keys by groups created on the basis of drug of choice ((a) = crack/cocaine, *n* = 31; (b) = heroin, *n* = 4; and (c) = alcohol, *n* = 4). A progressive ratio schedule of reinforcement controlled the relationship between responses on the keys and a 1 sec activation of pictures. Four different keys generated images of cocaine/crack, heroin, marijuana, or alcohol look-alike substances use, and paraphernalia. Two additional keys generated control images of buildings and random colors. The ∗ indicates a significant difference, within group, between responding a specific key and all other keys. In (a) responses to the crack/cocaine and heroin keys were not significantly different from each other.

Time spent on the CRT task varied between approximately 3 and 5 minutes. From a conditioning perspective, it was predicted that specific images of drugs/drug paraphernalia/drug-taking behavior would serve as reinforcers primarily in those subjects who identified that drug as their drug of choice. The results of the CRT partially supported this prediction. In fact, the crack/cocaine group emitted significantly more responses on the keys generating images of crack/cocaine and heroin (in comparison to control images—Figure 2(a); [*X* ^2^(5) = 25.04, *P* = 0.0001]), the heroin group emitted more responses on the key generating heroin images (in comparison to control images—Figure 2(b)), but the alcohol group showed no apparent response bias (Figure 2(c)). 

**Figure 3:**
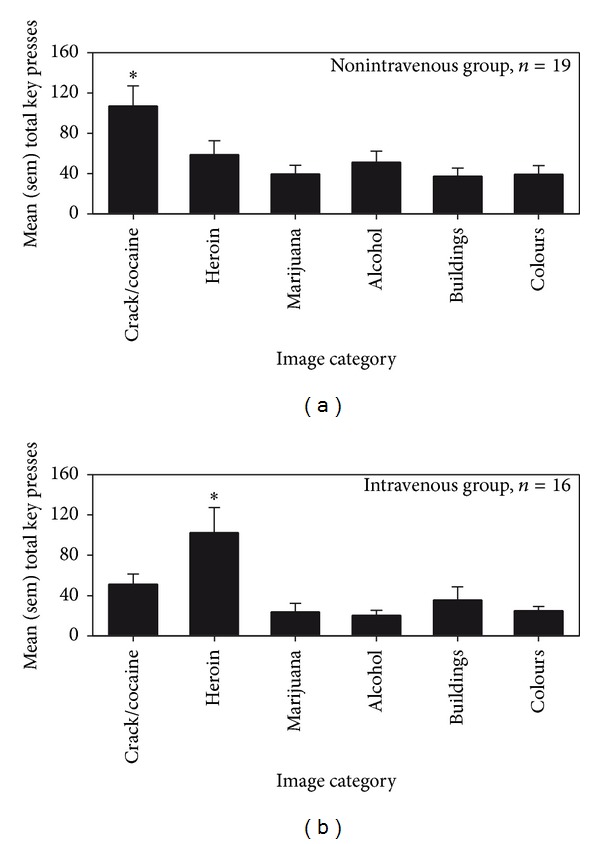
CRT performance of volunteers tested at Stonehenge Therapeutic Community. Mean and sem total number of responses made on computer keys by groups created on the basis of route of administration ((a) = nonintravenous (smoking/snorting), *n* = 19; (b) = intravenous, *n* = 16). The ∗  indicates a significant difference, within group, between responding a specific key and all other keys.

When considering the interpretations of these results, it was noted that many subjects who reported crack/cocaine as drug of choice also reported intravenous use, and that images of needles and injection/injection rituals were included only in the “heroin” category. Therefore, the subjects were re-grouped on the basis of preferred route of administration: nonintravenous (smoked and snorted), *n* = 19; intravenous, *n* = 16; and oral (drank alcohol), *n* = 4, and the analysis of responses was repeated. It was found that the non-intravenous group responded significantly more to the key generating images of powder/crack smoking and snorting paraphernalia in comparison to control images (“crack/cocaine” category in Figure 3(a); [*X* ^2^(5) = 20.79, *P* = 0.0008]). By contrast, the intravenous group responded significantly more to the key generating images of needle paraphernalia and intravenous usage in comparison to control images (“heroin” category in Figure 3(b); [*X* ^2^(5) = 13.74, *P* = 0.017]). Level of operant responding of the third group (oral) was already represented in Figure 2(c) (alcohol), and no differences were apparent.

**Figure 4:**
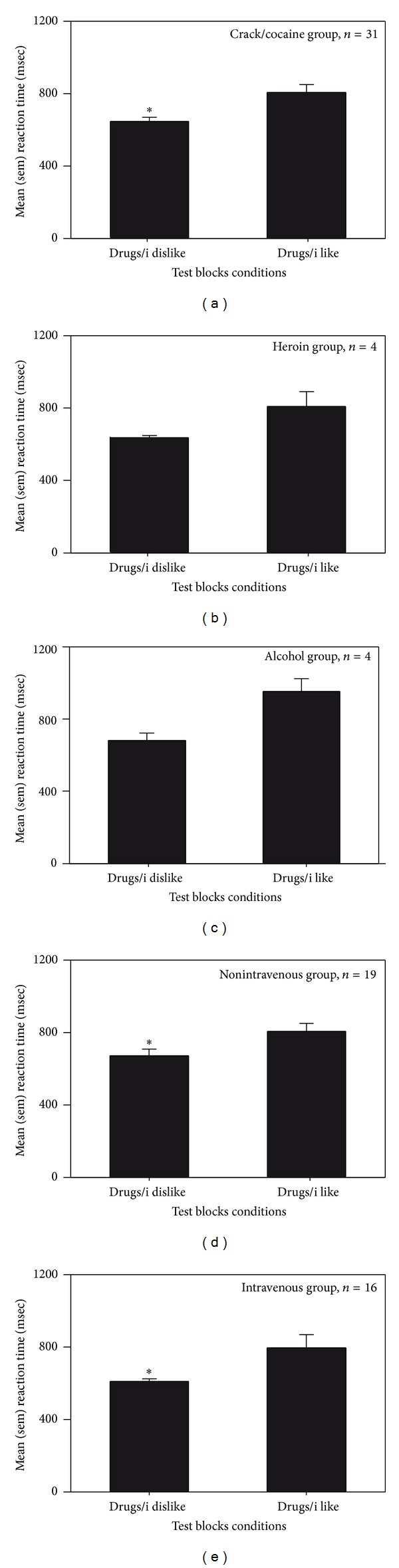
IAT performance of volunteers tested at Stonehenge Therapeutic Community. Mean and sem reaction times (msec) on test trials comparing “drugs” and “I dislike” versus “drugs” and “I like” in groups created on the basis of drug of choice or preferred route of administration ((a) = crack/cocaine, (b) = heroin, (c) = alcohol, (d) = non intravenous; and (e) = intravenous). The ∗ indicates a significant difference within group.

On the IAT, it was found that reaction times were quicker when the categories “drugs” and “I dislike” shared the same side of the computer screen, in comparison to when the same side of the screen was shared by the categories “drugs” and “I like.” This effect was equivalent when groups were created by drug of choice (Figure 4(a): crack/cocaine group [*t*(30) = 3.85, *P* = 0.0006]; heroin (Figure 4(b)) and alcohol (Figure 4(c)) groups: trend in the same direction) or by preferred route of administration (Figure 4(d): non-intravenous group [*t*(17) = −3.12, *P* = 0.006]; Figure 4(e): intravenous group [*W* = 74.00, *Z* = 2.90, *P* = 0.001]; (Figure 4(c)) oral group: trend in the same direction). Thus, overall, the IAT D scores were negative.

There were no significant correlations between craving scores, responding on the preferred key in the CRT (regardless of grouping), and IAT D scores. However, in the non-intravenous group, there was a significant negative correlation between days in treatment and responding on the key generating powder/crack smoking and snorting paraphernalia [*r* = −0.59, *P* = 0.0068; corrected *α* = 0.016]. Finally, when treatment completers (87%) and noncompleters (13%) were compared, no significant differences were found in responding to the preferred key in the CRT (regardless of grouping), craving scores, or IAT D scores. 

### 3.2. Alcohol Study

From the TFM questionnaire, it was established that the average number of days on which drinking occurred in the 30 days previous to the interview was 5.6 ± 0.5. In spite of infrequent drinking, more than half of the subjects had an ADS score equal or greater than 9 (see Table 1) and, overall, there was a significant positive correlation between ADS score and self-reported craving for alcohol (*r* = 0.40, *P* = 0.004). 

Unlike in participants tested at Stonehenge, the undergraduates in this study did not display a significant response bias on the CRT (time spent on the CRT task varied between approximately 3 and 4 minutes), regardless of the ADS score (see Table 1). However, as in the study at Stonehenge, reaction times on the IAT were significantly quicker when the categories “alcohol” and “I dislike” shared the same side on the screen (Table 2; <9 group: [*t*(41) = −2.96, *P* = 0.007]; ≥ 9 group: [*t*(26) = −2.67, *P* = 0.013]). Although there were no overall significant correlations between the IAT D scores and ADS scores, craving scores, or responding on the CRT, it was noted that 65% of the participants were females, and when ADS scores were correlated to IAT D scores and craving scores separately in females and males, it was found that, in males, higher ADS scores were associated with more positive automatic attitudes (*r* = 0.66, *P* = 0.005) and with higher craving scores (*r* = 0.49, *P* = 0.051). 

### 3.3. Sex Study

From the questionnaires about sexual behavior and sexual craving, it was noted that although males and females did not differ on craving (97.7 ± 4.1 and 92.7 ± 4.2), there was a significant modulation by relationship status. In fact, craving for sex was significantly higher in both males [*t*(41) = 2.39, *P* = 0.021] and females [*t*(61) = 2.49, *P* = 0.015] that were actively involved in a relationship at the time of testing (Table 2). This suggested that regular access to a sexual partner could play an important role in modulating performance on the CRT and IAT. Therefore, for analyses of performance on these tests, both males and females were further subdivided in those involved or not involved in a relationship (Table 2). 

On the CRT, both males and females responded significantly more to the key generating images of sexy women or men, compared to all other keys, respectively (Table 2; significant main effect of Key [*F*(5,510) = 11.02, *P* < 0.0001] and significant interaction between Gender and Key [*F*(5,510) = 29.53, *P* < 0.0001]; statement above based on the results of multiple comparisons). Time spent on the CRT task varied between approximately 3 and 4 minutes. Furthermore, within both males and females, those involved in relationships responded significantly more to activate images of models of the opposite sex (significant main effect of relationship status [*F*(1,102) = 11.97, *P* = 0.0008], significant interaction between relationship status, and key [*F*(5,510) = 3.61, *P* = 0.003], and significant interaction between gender, relationship status, and key [*F*(5,510) = 4.51, *P* = 0.0005]; statement above based on the results of multiple comparisons). Finally, there was a significant correlation between responses to view images of models of the opposite sex and craving scores (males and females combined; *r* = 0.43, *P* = 0.001), but only for those involved in a relationship. 

The analysis of IAT data revealed that both males and females were slower to respond when the categories “sex” and “I dislike” shared the same side of the screen, and this effect was not significantly altered by relationship status (males: main effect of category [*F*(1,39) = 9.99, *P* = 0.003]; females: main effect of category [*F*(1,54) = 12.74, *P* = 0.0008]). There were no significant correlations between the IAT D scores and craving scores or responding to the CRT. 

## 4. Discussion

The principal finding of this study is that individuals in long-term residential treatment for substance dependence emitted a significant number of operant responses (i.e., presses on a computer key) to view images of drugs, drug use, and drug paraphernalia. Responding was selective to images of drug of choice and of paraphernalia associated with participants' preferred route of administration. In fact, those reporting crack cocaine as their drug of choice responded significantly more on the key activating images of crack cocaine and crack cocaine use/pipes. And, when groups were re-established on the basis of typical route of administration, it was found that injectors responded preferentially to the key generating images of needles and associated paraphernalia/use, and smokers/inhalers responded preferentially to the key generating images of white power, crystals, and associated paraphernalia/use. 

It is widely believed that selective attention to drug related stimuli is critical for the experience of cravings and the maintenance of addictive behaviors, and it is known that users display attention biases for drug related words, scenes, and images [5, 7, 55]. For example, Moeller et al. [56] found that the choice of cocaine-related picture correlated with subjects' concurrent cocaine and other drug use, and predicted cocaine and other drug use over a period of 6 months. The results of the conditioned reinforcement task in abstinent participants corroborate and expand these findings. Not only did subjects voluntarily select the key(s) generating images of drug/route of choice while in treatment, but also responded more to these keys (versus other keys) in spite of progressively escalating response requirements. 

The primary interpretation of this finding is based on classical learning theory, which suggests that drug associated stimuli acquire conditioned reinforcing properties through association with the effects of drugs [57], and hence gain the ability to reinforce behavior in the absence of drugs [16]. However, there are possible alternative interpretations. For example, subjects may have been bored, and thus willing to respond to any novel image. That said, participants' responding was significantly greater on keys generating images directly associated with their drug of choice or preferred route of administration. Alternatively, the images of drugs and drug paraphernalia might have been very appealing, and thus capable of promoting responding independent of prior learning. However, this seems unlikely given that undergraduate students selected on the basis of alcohol use (Alcohol study) responded very little to keys generating images of powders, needles, crack pipes, or burning spoons. Finally, the conditioning interpretation is further supported by the findings of the sex study. In fact, participants' magnitude of responding to view pictures of sexy opposite-sex models was significantly modulated by the frequency of sexual behavior. Hence, more frequent contact with the unconditioned stimulus (in this case sexual partner) increased responses to stimuli predictive of sexual behavior (sexy models; conditioned stimuli). Therefore, although the alcohol and the sex studies were not performed in subjects that were matched to subjects in the Stonehenge studies for age, gender, race, and education, they provided important results about basic psychological processes activated by the exposure to learned incentive stimuli.

Interestingly, a different pattern of responding was observed in abstinent alcoholics in treatment at Stonehenge, who did not preferentially respond to the key generating images of beer, wine, spirits, and consumption of these beverages. Although it is possible that this was due to a low-sample size (*n* = 4), it should be noted that low response to these images was also observed in undergraduate students who scored above 9 on the ADS (see results of Alcohol study). The discrepancy between findings with alcohol and other drugs/sexual images is difficult to explain. It could be that there is something peculiar about alcohol or alcoholics [58, 59], although it is more likely that the images of alcohol/drinking were not specific enough [60] (i.e., preferred drink or brand). Such possibility could be tested by recruiting a larger sample and by presenting subjects with keys generating images of specific alcoholic beverages and then determine whether key selection is related to beverage of choice. 

In the study at Stonehenge, the lack of correlation between responding to the CRT and score on the drug craving questionnaire could imply that the psychological constructs assessed by these tasks are independent. Although it is possible that subjects may have not been willing to disclose their craving because of the therapeutic setting in which testing was conducted, it is more likely that the low craving scores may have resulted from perceived “nonavailability” of drugs [61–63]. Such interpretation is supported by the Sex study, in which it was found that self-reported levels of craving for sex were significantly higher in those in active relationships, and frequency of sexual behavior was significantly associated with responding to the CRT. Furthermore, in a separate pilot study (*n* = 18) also performed at the Stonehenge Therapeutic Community, craving was assessed before and after performance on the CRT, and the pre- and post-CRT craving scores were virtually identical. Therefore, it is likely that within the context of long-term treatment centers, craving may be a psychological dimension of substance dependence that is more difficult to assess using a questionnaire and/or manipulate by exposure to drug-associated stimuli. This is consistent with low levels of spontaneous craving described within inpatient addiction units for alcohol and cocaine dependence (see [64] for review).

Also, in the study at Stonehenge, it was found that the drug images employed in the CRT elicited significant negative automatic associations assessed by the personalized IAT. It is important to note that the IAT does not measure attitudes toward the exemplars (i.e., a specific picture of cocaine) but rather the concepts primed by the exemplars [65]. Furthermore, the specific task employed in the current study has been found to assess personal evaluative associations independent from cultural norms [46]. Therefore, this pattern of results suggests that conditioned stimuli can reinforce operant responding independently from their automatic valence, and that they retain the ability to generate these responses in abstinent individuals. 

Previous studies of automatic evaluations of alcohol using the personalized IAT revealed mixed findings. One study of light drinkers found significant negative implicit attitudes [66] and one study of heavy drinkers found a nonsignificant trend toward positive implicit attitudes [50]. Because negative implicit associations were also observed in student drinkers tested in the Alcohol study, it is possible that the image-based version of the personalized IAT does not explore the same automatic concepts that are generated by words (i.e., beer, wine). But, other explanations exist. First, students completing the image-based sex IAT generated scores reflective of positive automatic attitudes toward sex. Therefore, exposure to images can indeed activate positive evaluative reactions. Second, the personalized IAT has never been administered to substance dependent individuals recovering from cocaine and opiate addiction, and it is very likely that implicit attitudes toward these drugs change during the development of dependence. Finally, in the Alcohol study, ADS scores were associated with more positive automatic attitudes (*r* = 0.66, *P* = 0.005) and with higher craving scores (*r* = 0.49, *P* = 0.051), but in males only. The reason for the discrepancy between males and females in not clear, although it is fairly well established that there are significant sexual differences in psychological and physiological responses to alcohol [67–69]. 

Somewhat disappointing was the lack of significant relationship between performance on the CRT and treatment completion, even though duration in treatment was negatively correlated to amount of responding on the crack/cocaine key. Clearly, this issue should be addressed more systematically by additional studies that could administer the CRT at multiple times during treatment. And, it may be premature to dismiss the predictive clinical value of the CRT because it is also possible that self-selection bias played an important confounding role. In fact, approximately 87% of the individuals who volunteered for this study completed the program, and this is at odds with typical retention rates at Stonehenge of 40%–50%, which are in line with those of other therapeutic communities [70]. 

In conclusion, although the data should be considered preliminary in light of small group sizes, this paper reports that substance dependent individuals in a long-term residential treatment program who did not report significant cravings for drugs voluntarily responded to view images of preferred drugs/drug use or preferred route of administration. Although the predictive clinical utility of the CRT is yet to be fully validated, current treatment approaches based on cue-exposure and extinction [71] could profit from assessing behavioral responses to drug conditioned stimuli when self-reports of drug craving are uninformative.

## Figures and Tables

**Table 1:** Groups, sample size, craving score, and performance on the CRT and IAT in subjects tested in the *Alcohol study*.

ADS score	*n*	Craving	CRT						IAT	
			Crack/cocaine	Heroin	Marijuana	Alcohol	Buildings	Colors	Alcohol/I dislike	Alcohol/I like
<9	22	31.6 (4.1)	13.5 (3.4)	11.7 (2.8)	10.4 (2.1)	22.7 (6.8)	17.8 (3)	20.1 (4.1)	581.8 (19.8)*	676.1 (27)
≥9	27	42.8 (4.7)	14.5 (2.8)	11.2 (3)	14.7 (2.8)	18 (2.4)	17.1 (2.4)	23.4 (4.7)	567.6 (23)*	640.7 (23.4)

The first two columns include sample size and scores on the alcohol craving questionnaire in subjects scoring below, or equal to and above, 9 (i.e., threshold of potential problematic drinking) on the ADS. The next six columns include performance on the CRT (mean (sem) responses on each key). The last two columns include performance on the IAT (mean (sem) reaction time in msec). The ∗ indicates a significant difference within group.

**Table 2:** Groups, sample size, craving score, and performance on the CRT and IAT in subjects tested in the *Sex study*.

	*n*	Craving	CRT						IAT	
			Male model	Female model	“Junk” food	Snacks	Buildings	Colors	Sex/I dislike	Sex/I like
Males Involved	20	108 (6.5)*	7 (3.3)	429 (84.8)^∗#^	70.5 (20.2)	62.6 (16.6)	51.3 (15.7)	75.3 (18.6)	624 (32.1)	555.1 (25.1)*
Males not involved	23	89 (4.7)	4.5 (1.1)	155.3 (46.3)*	57.3 (13.6)	69 (20)	28.3 (8.4)	51.6 (15.1)	605.2 (25)	545.3 (21.3)*
Females involved	36	102 (5.4)*	265.3 (92)^∗#^	34.2 (13.6)	38.5 (9.5)	64.1 (11.7)	30.8 (8.8)	93 (23.7)	590.2 (16.8)	543.7 (13.1)*
Females not involved	27	81 (6.2)	165.8 (41)*	11.7 (3.2)	22.7 (4.3)	51.4 (11.3)	17.6 (3.4)	48.7 (11.2)	716.1 (49.3)	604 (23)*

The first two columns include sample size and scores on the sexual craving questionnaire. The ∗ indicates a significant difference, within gender, between those involved and those not involved in a relationship. The next six columns include performance on the CRT (mean (sem) responses on each key). The ∗ indicates a significant difference, within group, between responding a specific key and all other keys; the # indicates a significant difference in response on the same key, within gender, between those involved and not involved in a relationship. The last two columns include performance on the IAT (mean (sem) reaction time in msec). The ∗ indicates a significant difference within group.
